# Antibiotic Prophylaxis with Trimethoprim-Sulfamethoxazole versus No Treatment after Mid-to-Distal Hypospadias Repair: A Prospective, Randomized Study

**DOI:** 10.1155/2018/7031906

**Published:** 2018-03-26

**Authors:** Elizabeth B. Roth, John V. Kryger, Charles T. Durkee, Melissa A. Lingongo, Ruth M. Swedler, Travis W. Groth

**Affiliations:** Division of Pediatric Urology, Department of Urology, Medical College of Wisconsin, Children's Hospital of Wisconsin, Milwaukee, WI, USA

## Abstract

**Purpose:**

To evaluate the impact of prophylactic antibiotics after distal hypospadias repair on postoperative bacteriuria, symptomatic urinary tract infection, and postoperative complications in a prospective, randomized trial.

**Materials and Methods:**

Consecutive patients aged 6 months to 2 years were enrolled at our institution between June 2013 and May 2017. Consenting patients were randomized to antibiotic prophylaxis with trimethoprim-sulfamethoxazole versus no antibiotic. Patients had catheterized urine samples obtained at surgery and 6–10 days postoperatively. The primary outcome was bacteriuria and pyuria at postoperative urine collection. Secondary outcomes included symptomatic urinary tract infection and postoperative complications.

**Results:**

70 patients consented to the study, of which 35 were randomized to receive antibiotics compared to 32 who did not. Demographics, severity of hypospadias, and type of repair were similar between the groups. Patients in the treatment group had significantly less pyuria (18%) and bacteriuria (11%) present at stent removal compared to the nontreatment group (55% and 63%; *p*=0.01 and *p* < 0.001, resp.). No patient had a symptomatic urinary tract infection. There were 11 postoperative complications.

**Conclusions:**

Routine antibiotic prophylaxis appears to significantly decrease bacteriuria and pyuria in the immediate postoperative period; however, no difference was observed in symptomatic urinary tract infection or postoperative complications. Clinical Trial Registration Number NCT02593903.

## 1. Introduction

Hypospadias occurs in at least 1/300 live births [1], making it one of the most common entities treated by pediatric urologists. A 2010 report of practice patterns among pediatric urologists in North America showed that >90% place patients on routine antibiotic prophylaxis after single-stage hypospadias repair requiring use of a urethral stent [2]. As such, this represents a significant utilization of antibiotics in the pediatric urology practice. While antibiotic prophylaxis has been extensively studied in patients with other urologic conditions, including hydronephrosis and vesicoureteral reflux, less is known about the efficacy and necessity of antibiotic prophylaxis in the setting of hypospadias repair.

Three studies in the literature have previously examined this question [3–5] but reached different conclusions regarding the efficacy of prophylaxis. Meir and Livne showed support for prophylaxis, with increased bacteriuria and a clinical urinary tract infection rate of 24% in children who did not receive postoperative antibiotic prophylaxis as compared to those that did [3]. Conversely, a study by Kanaroglou et al. showed no difference in symptomatic urinary tract infections or postoperative complications between two retrospective cohorts of patients, one receiving antibiotic prophylaxis and one not receiving prophylaxis [4]. A more recent study in Sweden reported a higher complication rate (26% versus 15%) and a similar infection rate (5% versus 4%) in children receiving a decreased two-dose antibiotic course as compared to full-dose TMP-SMX in children who underwent tubularized incised plate repair [5]. To date, no published study has prospectively examined the efficacy of antibiotics at a prophylactic dose in the acute postoperative setting after single-stage primary repair of mid-to-distal hypospadias. Our study aims to address this question with regard to urine characteristics in the postoperative period as well as clinical outcomes of postoperative complications and symptomatic UTIs.

## 2. Materials and Methods

After approval by our institutional review board, we prospectively enrolled patients between 6 months and 2 years of age with mid-to-distal shaft hypospadias scheduled for elective primary hypospadias repair at our institution between June 2013 and May 2017. Children with proximal hypospadias or a history of previous hypospadias repair were excluded, as were children with allergies to trimethoprim-sulfamethoxazole (TMP-SMX). Children were randomized into treatment (TMP-SMX prophylaxis at 3 mg/kg trimethoprim daily) versus nontreatment. Simple randomization using a random number generator was used to assign patients to treatment versus nontreatment groups. Surgeons were not aware of the patient's randomization at the time of surgery, and the surgical technique was not standardized. Patients did not routinely receive IV antibiotics perioperatively, and the treatment group was instructed to begin TMP-SMX on the evening of surgery and continue until the evening prior to catheter removal in the clinic. Postoperative care was standardized for all patients utilizing the same 6 Fr Kendall-type hydrophilic urethral stent (Covidien Dover Hydrogel Coated Urethral Catheter 6 Fr #6006) and double diaper urinary drainage for 6–10 days following surgery. All children had urine samples obtained intraoperatively and at the first postoperative clinic visit prior to catheter removal. Patients in the treatment group were asked to return study medication at their first postoperative clinic visit, and residual TMP-SMX was measured to assess compliance with the regimen.

The postoperative urine sample was obtained by cleaning the catheter hub with alcohol followed by clamping the catheter for 15–30 minutes to allow an adequate amount of urine to collect in the bladder. The catheter hub was then reprepped with alcohol and urine obtained prior to catheter removal.

Our primary endpoints were bacteriuria and pyuria at the time of the first postoperative visit. We defined bacteriuria as the presence of >50,000 colony-forming units on postoperative urine culture. These were chosen as primary endpoints due to the wide range of symptomatic UTIs in the postoperative period after hypospadias repair in previous studies (0–24%) and due the subjective nature of determining positive symptoms in this nonverbal population [3–5]. Power calculations based on published symptomatic UTI after hypospadias repair in the literature estimated 160 patients, 80 in each study arm to achieve 80% power with 95% confidence. Power calculations based on our primary endpoints were not performed as the prevalence of bacteriuria and pyuria in this population is not well established. Secondary endpoints were symptomatic UTI, defined as fever, pain, or other clinical symptoms consistent with UTI along with a urine culture of >50,000 CFU, and postoperative complications. Positive cultures without symptoms were not routinely treated to avoid exposure of either group to additional antibiotics. Measured postoperative complications included wound infection, urethrocutaneous fistula, wound dehiscence, and meatal stenosis. Data analysis was completed using standard, 2-tailed univariate statistical tests with significance of *p* < 0.05. Continuous variables were assessed with Student's *t*-test, and categorical variables were assessed utilizing the Fisher exact test.

## 3. Results

**Figure 1:**
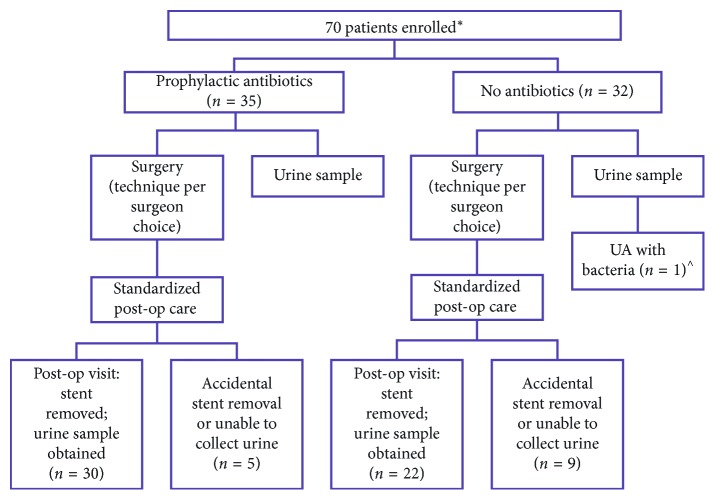
Study design. ^∗^3 patients withdrawn after enrolling in the study: 1 with proximal hypospadias on reexam at the time of surgery and 2 patients due to lack of pharmacy support to dispense the study drug if randomized to the treatment group. ^ The patient excluded from further analysis due to bacteriuria at surgery.

Of 113 consecutive eligible patients, 70 consented to the study. Thirty-five were randomized to receive TMP-SMX prophylaxis at 3 mg/kg daily, and 32 received no antibiotics. Three patients who enrolled in the study were unable to be randomized, one due to finding a proximal hypospadias during reexam under anesthesia at the time of operative repair and two due to lack of pharmacy support to provide the study drug should they be randomized to treatment with TMP-SMX. One patient in the nontreatment group had bacteria present on urinalysis in the OR and was excluded from further analysis (Figure 1).

Demographic and clinical data about hypospadias severity and type of repair were similar between both groups. Treatment group patients presented for surgery at 9.8 ± 3.1 months of age compared to 10.5 ± 4.7 months in the nontreatment group (*p*=0.45). Weight, presence of chordee, urethral deficit length, glans size, and type of repair were not significantly different between the two groups (Table 1). Study medication was returned by 31/35 patients, and 30 patients returned an amount consistent with >90% compliance with the prescribed regimen.

Postoperative urine samples were successfully obtained on 30/35 patients in the treatment group and 22/32 patients in the nontreatment group (*p*=0.14). Among the 15 patients unable to provide postoperative urinalysis or culture, 6 patients experienced premature catheter removal outside the clinic, 4 specimens were unable to be processed by the lab, 3 patients were unable to retain urine in the bladder during the clamping period, 1 parent refused specimen collection at postoperative visit, and 1 was excluded due to the presence of bacteria in urine at the time of surgery.

Leukocyte esterase was detected in 18% (5/28) of samples in the treatment group and 55% (11/20) of samples in the nontreatment group (*p*=0.01). Similarly, bacteriuria on urine microscopy was present in 11% (3/25) patients in the treatment group and 63% (7/19) of the nontreatment group (*p* < 0.001). Urine culture was positive for a single organism of at least 50,000 colony-forming units in 7% (2/30) of the treatment group and 64% (10/22) of the nontreatment group (*p* < 0.001). Organisms isolated from the cultures are reported in Table 2. Of 17 isolated organisms isolated from no treatment group tested against TMP-SMX, resistance was reported in 2/17 organisms (*E. coli* × 2) compared to 3/3 organisms (*E. coli* and *Enterococcus* species × 2) in the treatment group (*p*=0.009).

Postoperative complications were reported in 5/35 patients in the treatment group and 6/32 patients in the nontreatment group (*p*=0.75). Median follow-up of patients in the study was 25.5 months (2.4–39.9 months). Postoperative complications are summarized in Table 3. No patient in the study met the definition for a symptomatic UTI.

## 4. Discussion

While it remains common practice among pediatric urologists, the use of perioperative and postoperative antibiotics in hypospadias has not been well characterized in terms of postoperative urine studies or in terms of clinical outcomes. In the absence of true evidence-based guidelines, most providers rely heavily on tradition and on clinical pathways learned during residency and fellowship training when determining whether or not to give antibiotics in this situation.

Based on our data, prophylactic antibiotics do decrease bacteriuria and markers of inflammation within the urine in the acute postoperative period (Table 2). The exact role that urinary tract inflammation and bacterial colonization play in overall surgical outcomes remains unclear, since no patient in the study met criteria for a clinical UTI, and surgical complications were similar between the groups (Table 3). Inadvertent stent removal and failure to obtain the postoperative urine sample were more common in the nontreatment group, though the difference failed to meet statistical significance. While it may be due to random chance, it is possible that antibiotic use may be protective against stent-related complications. Conversely, TMP-SMX resistance was significantly higher when bacteriuria was present in the postoperative urine sample in the treatment group as compared to the nontreatment group.

Our study aims to add to the body of evidence regarding postoperative antibiotic prophylaxis in patients undergoing mid-to-distal shaft hypospadias repair. Review of the previous study by Meir and Livne demonstrates a higher rate of bacteriuria and higher rate of urinary tract infection in children not receiving postoperative antibiotics but did not show significant differences in noninfectious postoperative complication rates [3]. Two important methodological differences exist between the Meir and Livne study and our study. First, the patients in the Meir study routinely received intravenous antibiotics at the time of surgery and were admitted to the hospital for 24 hours after the procedure. Patients in our study did not routinely receive intraoperative antibiotics, based on more recent evidence that IV antibiotics may not confer benefit in these patients [6] and all surgeries were performed as an outpatient. Second, while the Meir regimen was termed prophylactic, it consisted of treatment-dose antibiotics (cephalexin three times daily) given prophylactically to prevent bacteriuria, rather than prophylactic dosing of antibiotics according to standards for vesicoureteral reflux as utilized in our study.

Kanaroglou et al. studied this issue in a more contemporary patient population in a retrospective manner and found no difference in postoperative outcomes or postoperative UTI [4]. Similar to our patient population, children received prophylactic dosing of antibiotic with TMP-SMX at 2 mg/kg daily or appropriate alternative for sulfa-allergic patients. Again, all patients received intraoperative IV antibiotics at the time of surgery. Urine studies were not performed in this cohort, making comparisons to previous literature difficult.

More recently, Zeiai et al. reported a cohort of 113 patients who underwent tubularized incised plate repair at a single institution. Patients received either twice daily oral TMP-SMX preoperatively and continued on this dose until stent removal or a single dose of IV TMP-SMX at surgery and an oral dose at catheter removal. While complications were decreased in the reduced antibiotic group, this did not achieve statistical significance. Approximately 20% of this cohort had penoscrotal hypospadias, and this subset was responsible for nearly 50% of the reported complications in the group. Again, urine studies and antibiotic-resistance patterns were not examined in this cohort [5].

Our study represents the first published study to report both postoperative urine characteristics and postoperative outcomes in a prospective cohort of patients randomized to treatment with 3 mg/kg/day of TMP-SMX versus no treatment. Additionally, our study is the first to report on postoperative prophylaxis in a series of patients who did not receive routine IV antibiotics at the time of surgery.

While symptomatic UTI and skin infection are certainly the gold standard of clinical outcomes in this population, they are rare events and their prevalence is not agreed upon in the literature. For example, the previous 3 studies on antibiotic prophylaxis reported 0%, 5%, and 24% UTI rates in children, not on continuous antibiotic prophylaxis [3–5]. This makes ensuring adequate power difficult using these two endpoints and makes designing a study to address this issue definitively challenging within a single institution.

Bacteriuria and pyuria are objective endpoints that give a glimpse into the effects of antibiotic prophylaxis on the urinary milieu during initial healing in the acute postoperative phase. We thus felt that these were excellent endpoints to determine the effect of antibiotics on the patients and also that it was an important point to correlate urinary findings with UTI/skin infection in the same patient population, as this has not been done previously with regard to antibiotic prophylaxis. Additionally, we are the first to report a difference in resistance patterns among bacteria cultured from children with or without prophylaxis after hypospadias repair.

Despite its prospective nature, our study is not without limitations. First of all, our sample size is relatively small, despite 40 months of enrolling patients at a tertiary pediatric center with 4 pediatric urologists. Only 50% of parents approached about the study consented to be enrolled. This mainly served to highlight many of the difficulties inherent in performing randomized, prospective studies in a pediatric surgical setting. As detailed by Vemulakonda and Jones [7], randomization is a challenge for both patients and pediatric providers in a perioperative setting due to the perception that it takes some of the control of the perioperative course away from both parties and serves to emphasize the randomness that may occur in postoperative outcomes. We also experienced difficulties maintaining patients on the defined study protocol, with 22% (15/67) of patients unable to provide a urine sample at postoperative follow-up, mostly due to inadvertent early removal of urethral stent or due to difficulty processing specimens in the lab. Additionally, while we report postoperative outcomes for UTI and complications in our patient population, our follow-up time is limited (median: 25.5 months) and our study is not sufficiently powered to detect small differences in complications between the two groups. Despite these limitations, we believe this study to be an important contribution to the literature in an area where evidence-based recommendations are currently lacking.

## 5. Conclusions

In a prospective, randomized study of prophylactic antibiotics versus no antibiotics in patients undergoing mid-to-distal hypospadias repair with postoperative urethral stent, patients treated with TMP-SMX at 3 mg/kg/day experienced significantly lower rates of bacteriuria and pyuria at 6–10 days postoperatively than patients not treated with antibiotics. Additionally, organisms cultured from children on prophylaxis were significantly more likely to be resistant to the prophylactic agent. Despite this significant difference, no clinical differences between symptomatic UTI and postoperative complications were observed between the groups. Although limited by sample size and protocol deviation, our study lends credence to the growing body of literature that suggests that postoperative prophylaxis may not improve clinical outcomes in patients undergoing uncomplicated single-stage mid-to-distal hypospadias repair. Further study, likely a multi-institutional prospective trial, is indicated to determine if these outcomes are truly similar over a longer period of time.

## Figures and Tables

**Table 1:** Patient and hypospadias characteristics and repair type in treatment and nontreatment groups.

	Treatment (*n*=35)	Nontreatment (*n*=32)	*p* value
Age (months)	9.8 (±3.1)	10.5 (±4.7)	0.45
Weight (kg)	9.2 (±1.3)	9.3 (±2.0)	0.68
Type of hypospadias			
Midshaft	2	2	0.90
Distal shaft	27	26	
Megameatus	6	4	
Chordee present	20/33	19/31	0.96
Preop testosterone	5	2	0.43
Type of surgery			
TIP repair	17	18	0.74
Matthieu	4	4	
MAGPI	7	4	
Pyramid repair	6	4	
Urethral advancement	1	2	
Meatus to the tip of glans (mm)	8.0 (±2.6)	9.4 (±2.6)	0.05
Glans size (mm)	13.9 (±3.1)	14.6 (±2.3)	0.36

**Table 2:** Postoperative urine characteristics.

	Treatment (*n*=28)	Nontreatment (*n*=22)	*p* value
Leukocyte esterase	18% (5/28)	55% (11/20)	**0.01**
Nitrites	7% (2/28)	40% (8/20)	**0.01**
Bacteria	11% (3/27)	63% (12/19)	**<0.001**
White blood cells	4% (1/27)	47% (9/19)	**<0.001**
Culture with >50K CFU	7% (2/28)	64% (14/22)	**<0.001**
Organisms	*E. coli* × 1 *Enterococcus* × 1 *Enterobacter* × 1	*E. coli* × 7 *K. oxytoca* × 3 *Enterobacter* × 3 *Enterococcus* × 1 *Pseudomonas* × 1 *S. aureus* × 2 Mixed × 2	

**Table 3:** Postoperative complications and symptomatic UTIs.

Complications	Treatment (*n*=35)	Nontreatment (*n*=32)
Any	5	6
Fistula	1	2
Glans dehiscence	1	1
Wound infection	2	1
Meatal stenosis	1	2
Symptomatic UTI	0	0

## References

[1] Snodgrass W., Wein A. J., Kavoussi L. R., Novick A. C. (2011). Hypospadias. *Campbell-Walsh Urology*.

[2] Hsieh M. H., Wildenfels P., Gonzales E. T. (2011). Surgical antibiotic practices among pediatric urologists in the United States. *Journal of Pediatric Urology*.

[3] Meir D. B., Livne P. M. (2004). Is prophylactic antimicrobial treatment necessary after hypospadias repair?. *Journal of Urology*.

[4] Kanaroglou N., Wehbi E., Alotay A. (2013). Is there a role for prophylactic antibiotics after stented hypospadias repair?. *Journal of Urology*.

[5] Zeiai S., Nordenskjold A., Fossum M. (2016). Advantages of reduced prophylaxis after tubularized incised plate repair of hypospadias. *Journal of Urology*.

[6] Baillargeon E., Duan K., Brzezinski A. (2014). The role of prophylactic antibiotics in hypospadias repair. *Canadian Urological Association Journal*.

[7] Vemulakonda V. M., Jones J. (2016). Barriers to participation in surgical randomized controlled trials: a qualitative study of key stakeholder perspectives. *Journal of Pediatric Urology*.

